# The preterm human milk microbiota fluctuates by postpartum week and is characterized by gestational age and maternal BMI

**DOI:** 10.1128/mbio.02106-23

**Published:** 2023-11-17

**Authors:** Evgenia Jen Filatava, Zhongmao Liu, Jiaojiao Xie, Dong-Binh Tran, Kun Chen, Noura El Habbal, George Weinstock, Yanjiao Zhou, Katherine E. Gregory

**Affiliations:** 1 Boston College, Chestnut Hill, Massachusetts, USA; 2 University of Connecticut, Storrs, Connecticut, USA; 3 State Key Laboratory for Diagnosis and Treatment of Infectious Disease, The First Affiliated Hospital, School of Medicine, Zhejiang University, Hangzhou, Zhejiang, China; 4 Pfiizer Inc, Groton, Connecticut, USA; University of Maryland, School of Medicine, Baltimore, Maryland, USA

**Keywords:** human milk, preterm infants, microbiome, enteral nutrition, gestational age, infant formula, postpartum period

## Abstract

Evidence suggests that the type of nutrition fed to preterm infants influences their intestinal microbiome and immunity. However, few studies have conducted a longitudinal analysis of the microbiota of mother’s own milk (MOM) and the factors shaping its composition. Furthermore, the microbiota of pasteurized donor human milk (PDHM) and infant formula have not been extensively investigated. Here, we examine the microbiota of 238 MOM, 30 PDHM, and 73 formula samples from a cohort of 72 preterm infants using 16S rRNA sequencing. We find differences in the microbial diversity and composition between nutrition types. Additionally, we demonstrate that the microbiota of MOM exhibits temporal fluctuations and has associations with several maternal factors. Lastly, we identify three microbiota community clusters within MOM—termed lactotypes—that have distinct taxonomic compositions and maternal factors. Collectively, our findings lay the foundation for exploring the relationship between the microbiota of nutrition and preterm infant health outcomes.

**IMPORTANCE:**

Despite a growing recognition that the type of nutrition received by preterm infants influences their intestinal microbiome and health outcomes, the microbiota of mother's own milk (MOM), pasteurized donor human milk (PDHM), and infant formula remain poorly characterized. In our study, we found that the structure of microbial communities, bacterial diversity, and relative abundances of specific genera were significantly different between MOM, PDHM, and formula. Additionally, our results suggest that the microbiota of MOM changes as a function of time and maternal factors. Lastly, we identified three lactotypes within MOM that have distinct microbial compositions and described the maternal factors associated with them. These findings set the stage for future research aimed at advancing our knowledge of the microbiota of preterm infant nutrition and the specific influence it may have on health outcomes.

## INTRODUCTION

Mother’s own milk (MOM) is considered the optimal source of infant nutrition, promoting healthy growth and development throughout early childhood (1, 2). In addition to providing critical macro- and micronutrients, MOM contains many bioactive components (e.g., immunoglobulins, growth factors, and human milk oligosaccharides) that offer an array of health benefits, most notably a lower rate of necrotizing enterocolitis and sepsis in preterm infants (3, 4). Research has also shown that MOM contains a complex microbiota that may play a vital role in health promotion and disease prevention by modulating gut colonization and innate immunity during early life (5).

Despite a low microbial biomass, MOM is characterized by a diverse bacterial community with over 500 species identified (6). Nonetheless, a core milk microbiota consisting of a small number of species is often reported (7–14). The major hypotheses regarding the origins of bacteria in MOM have included maternal sources (i.e., mammary gland colonization via entero/oro-mammary routes or resident breast microbiota) and exogenous sources (i.e., intra-mammary milk inoculation via retrograde transfer from infant’s mouth or maternal skin, or contamination introduced during handling) (15–18). The microbiota of MOM has been found to exhibit both intra- and inter-individual variability as a function of gestational age (GA), mode of birth, antibiotic exposure, diet, body mass index (BMI), and geographic location, although the consequences of these differences in relation to infant health outcomes are not fully understood (19). Most studies profiling the microbiota of MOM have been cross-sectional, focused on early lactation, and limited with respect to elucidating the role of maternal factors, especially among women who give birth to preterm infants. A better understanding of the relationship between maternal factors and the microbiota of MOM is critical to advancing our knowledge of the mechanisms behind the protective effects of MOM and developing nutritional interventions aimed at improving preterm infant health outcomes.

If MOM is insufficient or unavailable, the early enteral nutrition (EN) needs of infants weighing under 1,500 g are met with pasteurized donor human milk (PDHM) or preterm infant formula. Unlike MOM, PDHM is thought to be virtually free of bacteria as a result of undergoing Holder pasteurization, which has been shown to kill or inactivate most microorganisms under research conditions (20). However, little is known about what, if any, microbiota exists within PDHM that is processed by milk banks for clinical use. Liquid infant formulas are considered sterile following ultra-high-temperature sterilization or retort sterilization. While these methods are widely accepted as effective in eradicating all bacteria, little is known about sequence-based bacteriologic surveillance of formula.

Evidence suggests that the intestinal microbiome and health outcomes of preterm infants are influenced by the type of EN they consume during early life (3, 21). Therefore, characterization of the microbiota of EN and, in the case of MOM, examination of maternal factors and temporal fluctuations at developmentally important time points, are critical in better understanding preterm infant health and disease. Thus, the aims of our observational study were to (i) describe and compare the microbiota of three EN types, (ii) examine temporal fluctuations in the microbiota of MOM, (iii) evaluate associations between the microbiota of MOM and maternal factors, and (iv) identify distinct microbial clusters within MOM (termed lactotypes) and the maternal factors associated with them.

## RESULTS

### Study population

**Fig 1:**
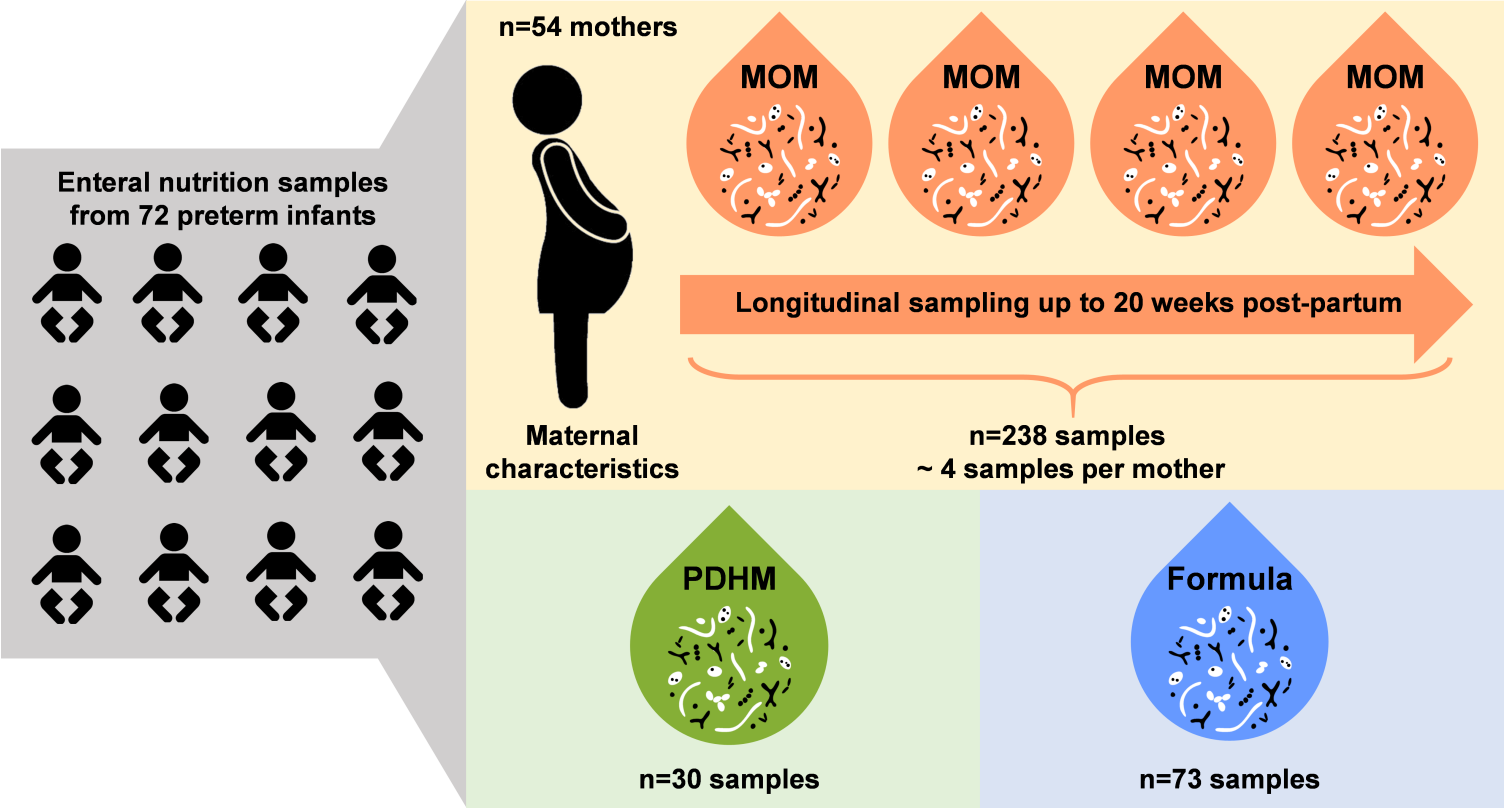
Study schema. Our study population included 65 mothers with singleton and twin births resulting in 72 infants. A total of 341 EN samples were used in our analyses: 238 MOM (83 MOM and 155 MOM + nutritional fortifiers [NF]), 30 PDHM (6 PDHM and 24 PDHM + NF), and 73 formula. The analyses that were limited to only MOM and MOM + NF samples were collected from 54 mothers in PPW 1–20 (238 samples) or in PPW 1–12 (222 samples). PPW, postpartum week.

The study population comprised 65 mothers with singleton and twin births resulting in 72 infants. The mean gestational age at birth was 29.5 ± 2.5 weeks. The detailed characteristics of the cohort are summarized in Table 1. A total of 341 EN samples (238 MOM, 30 PDHM, and 73 formula) were included in the analyses (Fig. 1).

**TABLE 1:** Maternal demographic and clinical factors

Maternal factors (*n* = 65)^*a*^	Mean ± SD or *n* (%)
Race	
White	33 (51%)
Black or African American	13 (20%)
Asian	6 (9%)
Unknown or not reported	13 (20%)
Ethnicity	
Not Hispanic or Latina	55 (85%)
Hispanic or Latina	10 (15%)
Age at time of birth (years)	32.9 ± 5.3
BMI at time of birth	31.0 ± 7.0
Primigravida	29 (45%)
Previous preterm birth	19 (29%)
Singleton birth	58 (89%)
Cesarean birth	50 (77%)
Gestational age at birth (weeks)	29.5 ± 2.4
PPW at time of MOM expression (weeks)	5.4 ± 3.9
PMA at time of MOM expression (weeks)	34.4 ± 3.5
ROM prior to birth	33 (51%)
Latency period after ROM (hours)	136.3 ± 300.7
Antenatal steroids	59 (91%)
Early antibiotics exposure (within ± 3 days of birth)	62 (95%)
Early cefazolin	48 (74%)
Early azithromycin	20 (31%)
Early penicillin	21 (32%)
Early other	20 (31%)
Late antibiotics exposure (>3 days postpartum)	16 (25%)
Maternal comorbidities	
Preeclampsia/eclampsia	13 (20%)
Chronic hypertension	8 (12%)
Diabetes (GDM or type 2)	12 (18%)
Previous tobacco use	8 (12%)
Completed DSQ	32 (49%)
Dairy daily intake (cup eq)	1.7 ± 0.6
Fruits daily intake (cup eq)	1.2 ± 0.4
Vegetables daily intake (cup eq)	1.5 ± 0.3
Added sugar daily intake (tsp eq)	17.6 ± 3.5
Whole grains daily intake (oz eq)	0.7 ± 0.3
Fiber daily intake (g)	18.5 ± 3.9
Calcium daily intake (mg)	929.5 ± 169.5

^*a*^ BMI, body mass index; PPW, postpartum week; PMA, postmenstrual age; ROM, rupture of membranes; GDM, gestational diabetes mellitus; DSQ, dietary screener questionnaire.

### Enteral nutrition microbiota differs by type

**Fig 2:**
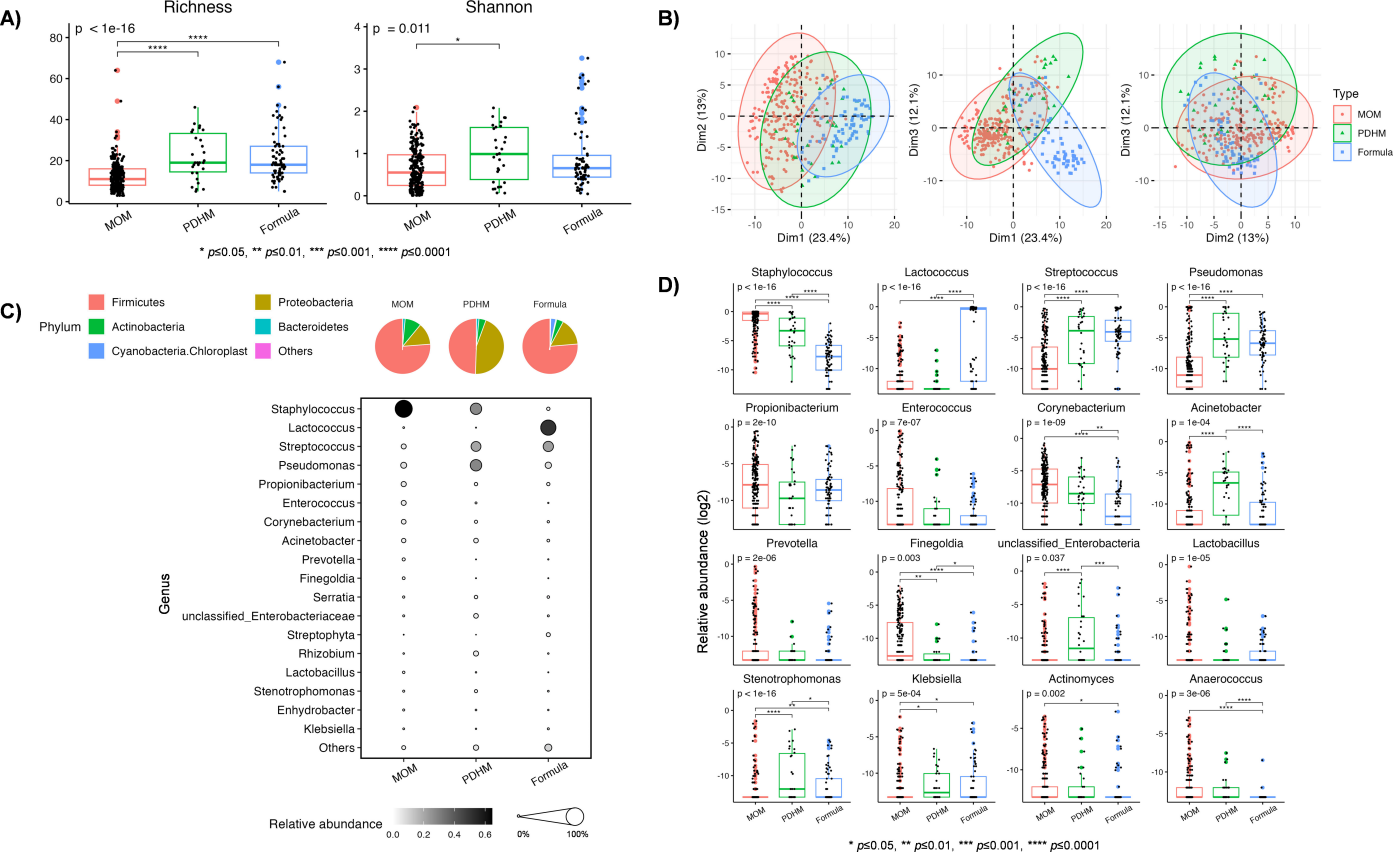
Comparison of diversity metrics and taxonomic composition by enteral nutrition type. (A) Alpha diversity (richness and Shannon), (B) PCA of beta diversity, (C) visualization of relative abundance of dominant bacterial phyla (pie chart) and genera (bubble plot), and (D) differential abundance of bacterial genera. formula, infant formula.

We observed significant differences in alpha diversity (richness *P* < 0.0001, Shannon *P* = 0.011) across EN types (Fig. 2A). Interestingly, MOM had a lower richness (*P* < 0.0001) compared to both PDHM and formula and a lower Shannon diversity (*P* < 0.05) compared to PDHM. Principal component analysis (PCA) analysis showed a clear separation between MOM, PDHM, and formula along the first component (Fig. 2B). Further analysis using permutational multivariate analysis of variance (PERMANOVA) revealed significant differences in beta diversity between PDHM and formula (*R*^2^ = 16.1%, *P* = 0.01) (Fig. 2B). We did not differentiate MOM vs PDHM or MOM vs formula using PERMANOVA because the homogeneity assumption was not satisfied.

Next, we sought to describe the bacterial composition of each EN type (Fig. 2C). At the phylum level, MOM was dominated by Firmicutes (76.2%), followed by Proteobacteria (12.8%), and Actinobacteria (9.5%). Formula was similarly dominated by Firmicutes (76.3%), followed by Proteobacteria (15.8%), Actinobacteria (4.0%), and Cyanobacteria (2.9%). In contrast, PDHM had similar abundances of Firmicutes (49.5%) and Proteobacteria (44.9%), followed by Actinobacteria (4.1%). At the genus level, MOM was dominated by *Staphylococcus* (64.6%), followed by *Pseudomonas* (6.6%). In contrast, PDHM had similar proportions of *Pseudomonas* (27.9%), *Staphylococcus* (27.6%), and *Streptococcus* (20.3%). Unlike MOM and PDHM, formula had a high abundance of *Lactococcus* (50.9%), followed by *Streptococcus* (22.1%), and *Pseudomonas* (7.5%).

**Fig 3:**
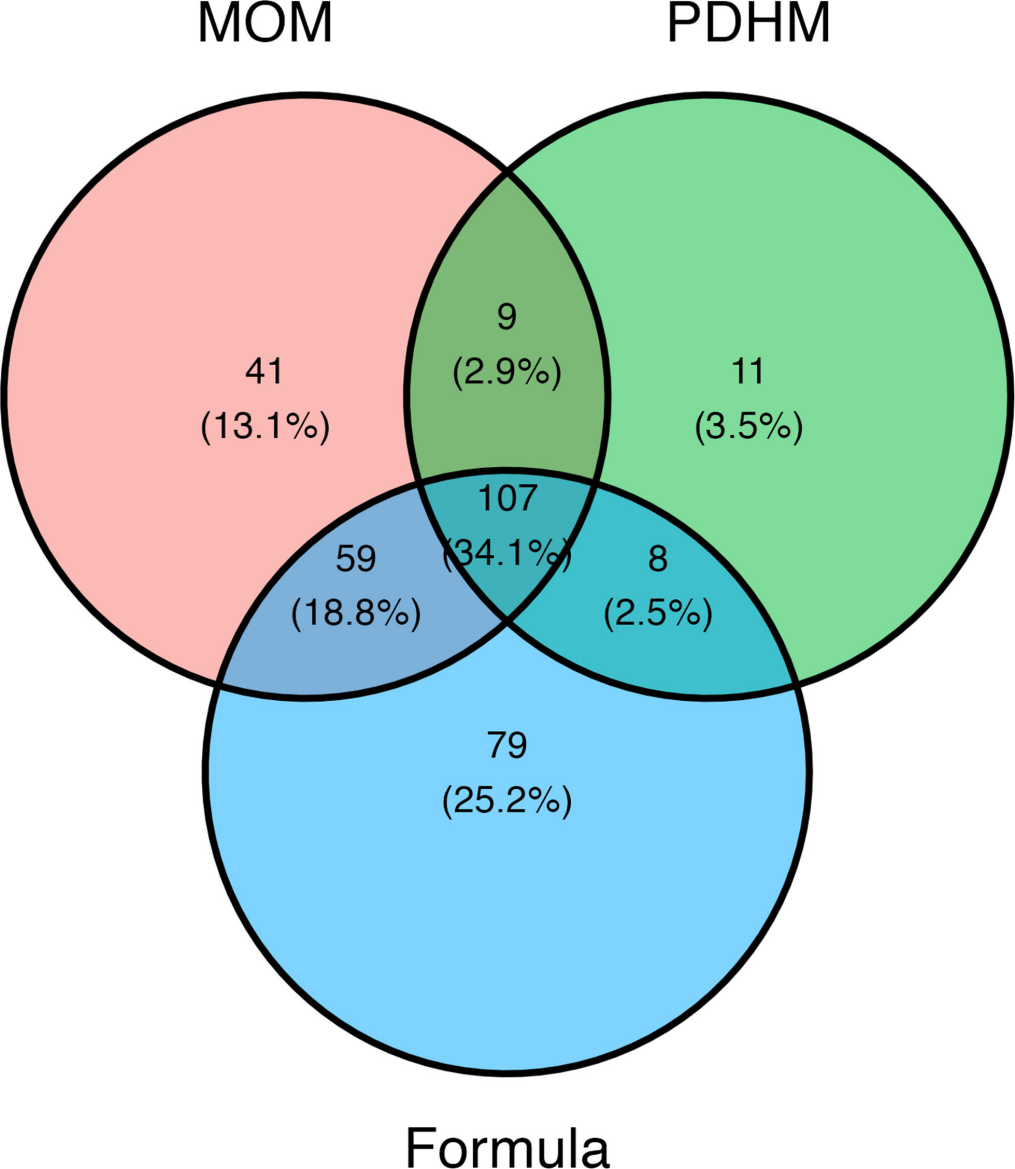
Venn diagram showing unique and shared genera between MOM, PDHM, and infant formula samples.

Of the 20 dominant genera, 16 exhibited differential abundance between EN types: *Staphylococcus* (*P* < 0.0001), *Lactococcus* (*P* < 0.0001), *Streptococcus* (*P* < 0.0001), *Pseudomonas* (*P* < 0.0001), *Propionibacterium* (*P* < 0.0001), *Enterococcus* (*P* < 0.0001), *Corynebacterium* (*P* < 0.0001), *Acinetobacter* (*P* = 0.0001), *Prevotella* (*P* < 0.0001), *Finegoldia* (*P* = 0.003), unclassified *Enterobacteria* (*P* = 0.037), *Lactobacillus* (*P* < 0.0001), *Stenotrophomonas* (*P* < 0.0001), *Klebsiella* (*P* = 0.0005), *Actinomyces* (*P* = 0.002), and *Anaerococcus* (*P* < 0.0001) (Fig. 2D). The number of common and unique genera found across EN types is summarized in Fig. 3.

### Milk microbiota changes over lactation

**Fig 4:**
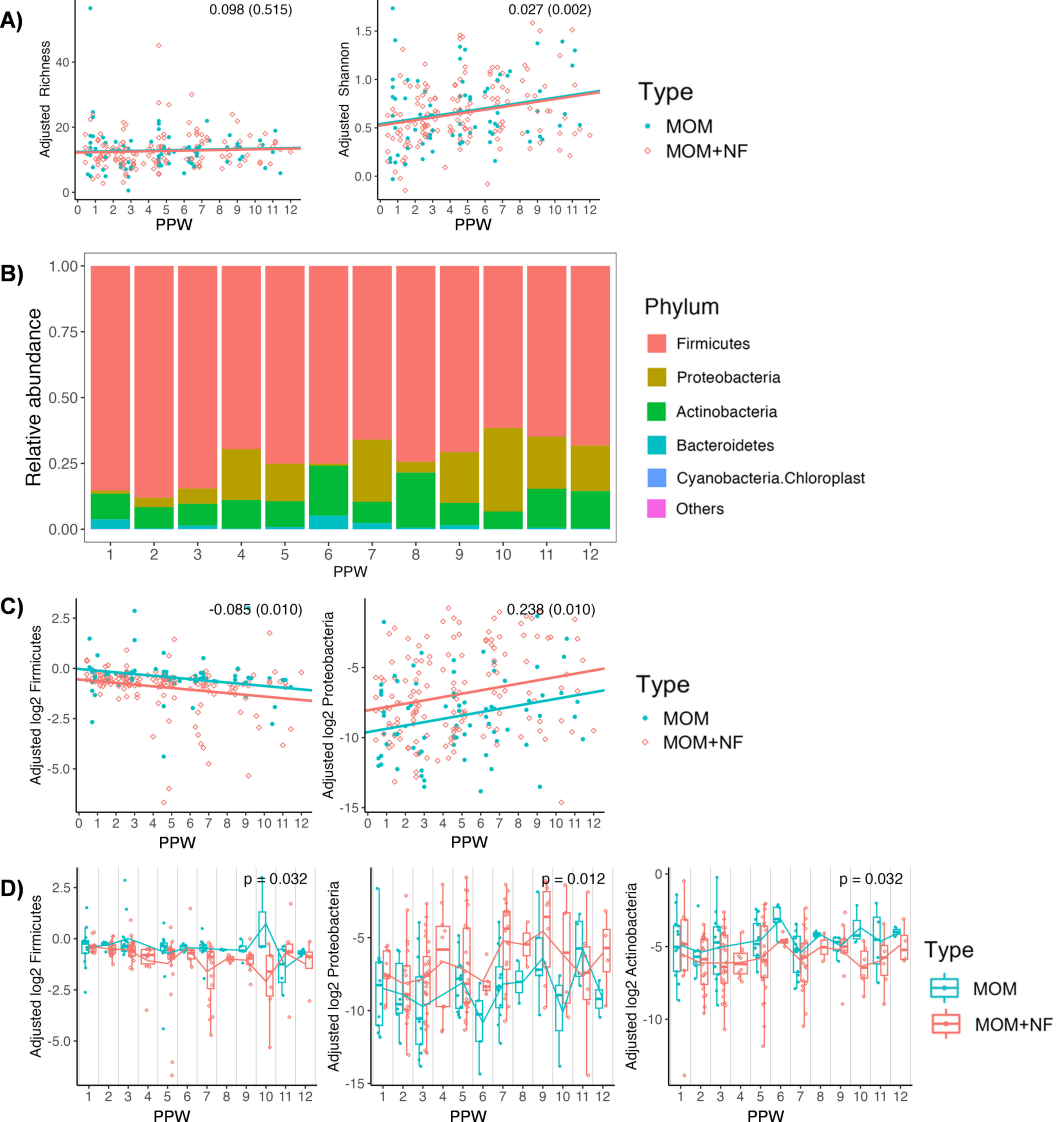
Changes in alpha diversity and phylum-level composition in MOM and MOM + NF over time. (A) Richness and Shannon, (B) visualization of relative abundance of dominant phyla over PPW (MOM and MOM + NF combined), (C) trend in phylum relative abundance over PPW, and (D) changes in phylum relative abundance at each PPW.

Next, we analyzed longitudinal trends in alpha diversity and bacterial composition in MOM and MOM + NF (MOM with nutritional fortifiers) during postpartum weeks (PPW) 1–12. Samples from PPW >12 were excluded due to an insufficient number of observations. We found a significant increase in Shannon diversity as a function of PPW in both MOM and MOM +NF (β = 0.027, *P* = 0.002), while richness remained stable (β = 0.098, *P* = 0.515) (Fig. 4A). Temporal changes in the phylum-level composition of MOM and MOM + NF (combined) are illustrated in Fig. 4B. In both groups, PPW was negatively associated with Firmicutes (β = −0.085, *P* = 0.010) and positively associated with Proteobacteria (β = 0.238, *P* = 0.010) (Fig. 4C).

**Fig 5:**
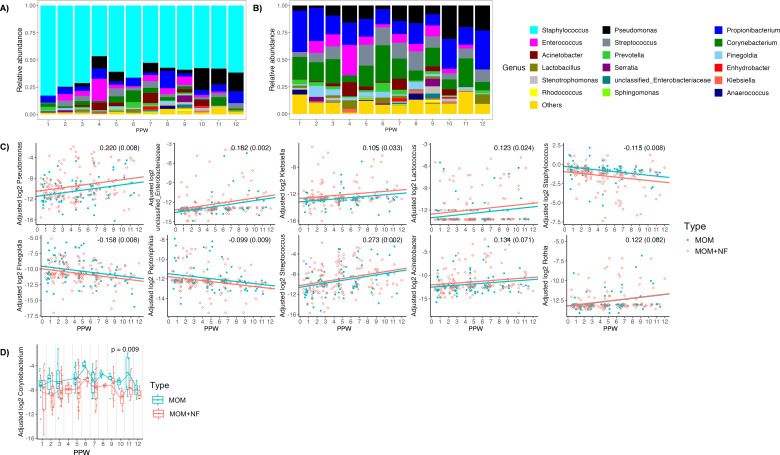
Changes in genus-level composition in MOM and MOM + NF over time. (A) Visualization of relative abundance of dominant genera over PPW (MOM and MOM + NF combined) with *Staphylococcus* and (B) without *Staphylococcus*, (C) trend in genera relative abundance over PPW, and (D) changes in genera relative abundance at each PPW.

Due to the high abundance of *Staphylococcus* in MOM and MOM + NF, we plotted the abundances of major genera with and without *Staphylococcus* for better visualization (Fig. 5A and B). We found that *Pseudomonas* (β = 0.220, *P* = 0.008), unclassified *Enterobacteria* (β = 0.182, *P* = 0.002), *Klebsiella* (β = 0.105, *P* = 0.033), *Lactococcus* (β = 0.123, *P* = 0.024), *Streptococcus* (β = 0.273, *P* = 0.002), *Acinetobacter* (β = 0.134, *P* = 0.071), and *Rothia* (β = 0.122, *P* = 0.002) increased over time, while *Staphylococcus* (β = −0.115, *P* = 0.008), *Finegoldia* (β = −0.158, *P* = 0.008), and *Peptoniphilus* (β = −0.099, *P* = 0.009) decreased over time in both groups (Fig. 5C).

To test whether the microbiota differed between MOM and MOM + NF during PPW 1–12, we fitted another model with PPW as a categorical fixed effect. At the phylum level, the abundance of Firmicutes, Proteobacteria, and Actinobacteria was significantly different between groups across all PPW (*P* = 0.032, *P* = 0.012, and *P* = 0.032, respectively) (Fig. 4D). At the genus level, the abundance of *Corynebacterium* was significantly different between groups across all PPW (*P* = 0.009) (Fig. 5D). The effect of group could not be tested in PPW 4 since there were only MOM + NF samples.

### Milk microbiota is associated with maternal factors

**Fig 6:**
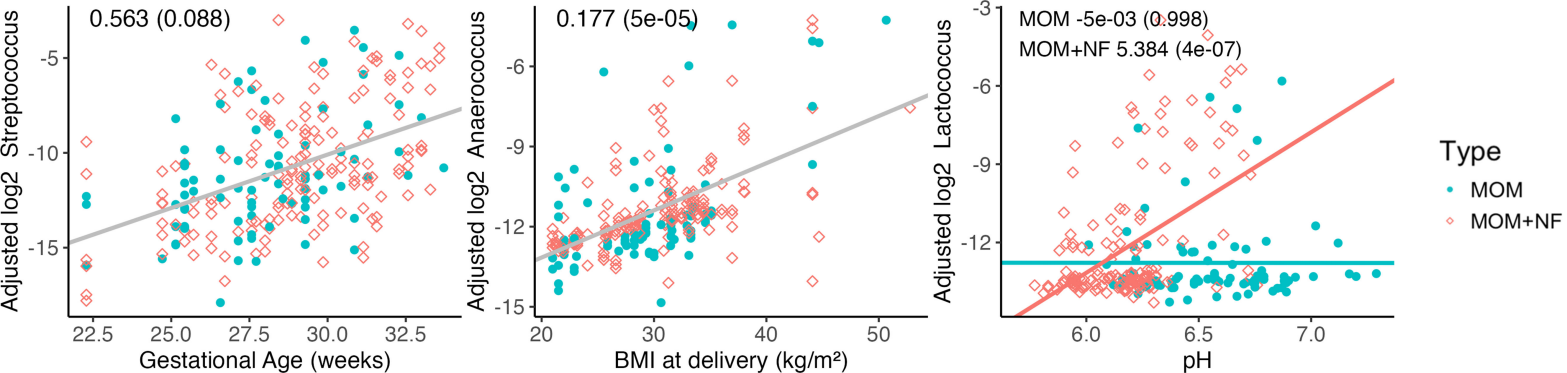
Associations between maternal factors and bacterial genera found in MOM and MOM + NF.

Next, we analyzed associations between maternal factors (Table 1) and the dominant genera found in MOM and MOM + NF. We identified a positive relationship between *Streptococcus* and GA at birth (β = 0.563, *P* = 0.088), *Anaerococcus* and maternal BMI at birth (β = 0.174, *P* = 0.00005), and *Lactococcus* and pH of MOM + NF (β = 5.384, *P* < 0.0001) (Fig. 6).

### Milk microbiota clusters into distinct lactotypes, which differ by maternal factors

**Fig 7:**
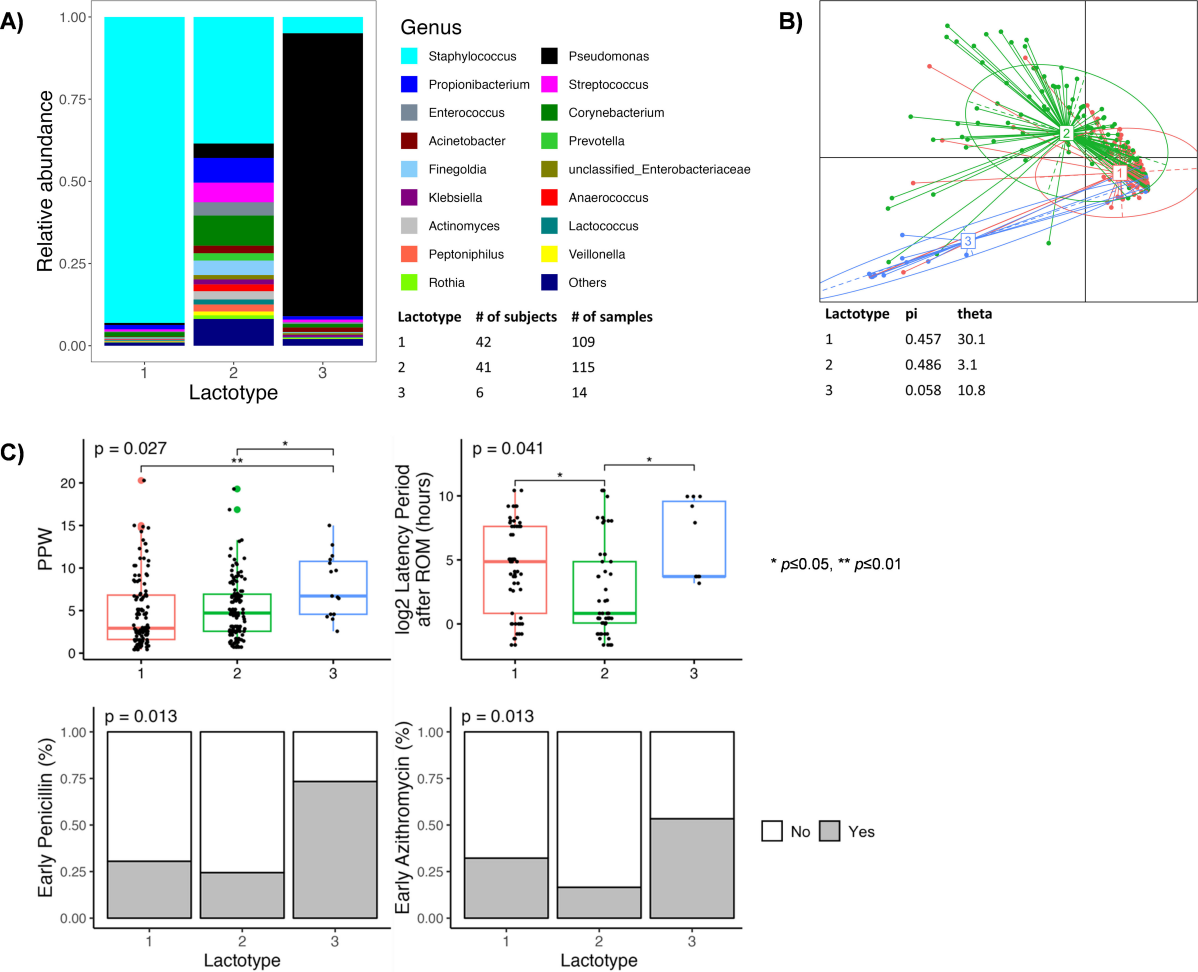
Taxonomic composition of lactotypes and comparison with maternal factors. (A) Visualization of relative abundance of dominant genera in each lactotype, (B) principal coordinate analysis of lactotypes, and (C) lactotypes as a function of maternal factors.

Because we observed dynamic changes in the bacterial composition of MOM and MOM + NF, we sought to test whether there are unique community clusters within these samples—termed lactotypes. Our analyses revealed three predominant lactotypes, each exhibiting a distinct taxonomic composition and bacterial abundances (Fig. 7A and B). Lactotype 1 (109 samples) was dominated by *Staphylococcus* (93.2%), while lactotype 3 (14 samples) was dominated by *Pseudomonas* (87.3%). In contrast, lactotype 2 (115 samples) was characterized by a more mixed composition of *Staphylococcus* (46.5%), *Enterococcus* (8.1%), *Propionibacterium* (7.7%), and *Streptococcus* (7.7%). The detailed taxonomic composition of lactotypes is provided in Table S1.

We then conducted a correlation analysis between lactotypes and maternal factors, with significant associations depicted in Fig. 7C. Interestingly, lactotypes differed by PPW (*P* = 0.027), with lactotype 3 tending to appear in later PPW compared to lactotype 2 (*P* < 0.05) and lactotype 1 (*P* < 0.01). Additionally, lactotypes differed by the latency period after rupture of membranes (ROM) (*P* = 0.041), with lactotype 2 tending to have a shorter latency period after ROM compared to lactotype 1 (*P* < 0.05) and lactotype 3 (*P* < 0.05). Lastly, lactotypes differed by exposure to azithromycin and penicillin (*P* = 0.013), where lactotypes 1 and 2 were less likely to have been exposed than lactotype 3.

## DISCUSSION

Research characterizing human milk microbiota has been largely focused on discrete points in time during early lactation among women with term-born infants. Here, we have focused on women with preterm-born infants, for whom early-life nutrition is especially critical to health outcomes. Initially, we aimed to describe and compare the microbiota of EN types as we are not aware of any studies to date that have done this. Then, we explored how the microbiota of MOM changes during the first 3 months of lactation. Lastly, we examined whether any maternal factors were associated with the microbial composition of MOM and whether the microbiota of MOM exhibited any clustering patterns (lactotypes). We acknowledge that our study was exploratory and that our findings should be interpreted with caution as our modest sample size reduced statistical power. Nonetheless, our work contributes to the limited but growing literature on the microbiota of EN and may guide future research efforts related to optimizing preterm infant nutrition.

When comparing the microbiota across EN types, we detected significant differences in alpha diversity with 16 genera exhibiting differential abundance between MOM, PDHM, and formula. It should be noted that our methods did not discern between alive and dead bacteria, and thus, our sequencing data should be interpreted as a reflection of both viable and non-viable bacteria. Our rationale for analyzing PDHM and formula, which are expected to contain a high proportion of dead bacteria due to pasteurization and sterilization, respectively, was based on recent evidence, suggesting that non-viable bacteria (e.g., postbiotics and paraprobiotics) may still influence host health (22). Additionally, culture-based studies have shown the presence of some viable bacteria in PDHM despite pasteurization (20, 23–25). Therefore, our goal was to identify all microbial DNA present in EN, irrespective of viability. With respect to formula samples, we acknowledge the possibility that the differences in the nutritional composition and manufacturing processes in the various formula types may influence the microbial composition in the sample and, in turn, in the infant gut. Future work may be well positioned to explore these differences and, in particular, could examine nutritional factors, such as caloric density, macronutrients, micronutrients, and bioactive components present in current and next-generation formulas.

Consistent with studies by Beghetti et al. and Mallardi et al., MOM had a lower alpha diversity than PDHM (14, 26). However, this is in contrast to the study by Cacho et al., who reported a similar alpha diversity between preterm MOM and PDHM (23). At the phylum level, MOM was dominated by Firmicutes and only had a small proportion of Proteobacteria, which is in line with the findings reported by Mallardi et al. (26). Similarly, in a study that combined term and preterm MOM, Firmicutes were more abundant than Proteobacteria (27). Jost et al. also reported a slightly higher abundance of Firmicutes than Proteobacteria in MOM from mothers who gave birth at term (28). Conversely, several studies reported higher proportions of Proteobacteria vs Firmicutes (13, 29–31). However, these studies used term MOM, and thus, comparisons with our results are limited.

Unlike MOM, PDHM had similar proportions of Firmicutes and Proteobacteria, which are consistent with the results reported by García-González et al. (32). Conversely, Mallardi et al. reported that PDHM consisted almost exclusively of Proteobacteria and very little Firmicutes (26). The 10 most abundant genera detected in our MOM samples (i.e., *Staphylococcus, Pseudomonas, Propionibacterium, Enterococcus, Streptococcus, Corynebacterium, Acinetobacter, Prevotella, Finegoldia,* and *Lactobacillus*) have all been previously reported as part of the milk microbiota, although with varying frequencies (33). Not surprisingly, MOM had the highest abundance of *Staphylococcus*, which is a well-known predominant member of the human milk microbiota, including preterm milk (6, 23, 27, 34, 35). Since *Staphylococcus* is commonly found in the hospital environment and on the skin of hospitalized preterm infants, Asbury et al. have proposed that its high abundance in preterm milk may be due to maternal exposure to the hospital environment and the skin-to-skin contact with the infant (34). Unlike MOM, PDHM did not have a single dominant genus and instead had similar proportions of *Pseudomonas*, *Staphylococcus*, and *Streptococcus*. According to a systematic review, *Pseudomonas* and *Streptococcus* are among the most frequently found genera in human milk after *Staphylococcus,* and their presence has also been reported specifically in PDHM (14, 19). *Bacillus*, which can survive pasteurization owing to its ability to release heat-resistant spores, was present in seven PDHM samples but at very low abundances. It is possible that milk banks screen and discard PDHM with higher levels of *Bacillus* due to its pathogenic potential.

We hypothesize that differences in alpha diversity and taxonomic composition between MOM and PDHM may be driven by differences in gestational age, time postpartum, and breastfeeding status. Although we do not have these data for PDHM samples, it is likely that they have more variability since PDHM is pooled from multiple donors, and each donor may have a unique combination of clinical factors. In contrast, our MOM samples were more uniform since all of them were collected from women with preterm births (<34 weeks GA) who expressed milk up to 5 months postpartum, and most of whom were unable to breastfeed due to infant’s prematurity. It is also possible that mothers of preterm infants followed more stringent hygiene practices when expressing their milk compared to mothers of term infants. Lastly, since PDHM is subjected to additional handling by milk banks, it is possible that some bacteria represent environmental contamination. Regardless of how PDHM is colonized, our samples were collected from what was provided to preterm infants, ensuring that our data reflect the microbial DNA present in PDHM at the time of feeding.

To our knowledge, this is the first study to report the microbial composition of infant formulas used in a neonatal intensive care unit (NICU). Contrary to our expectations, formula had a higher richness than MOM despite being sterilized. Interestingly, the proportions of Firmicutes and Proteobacteria were similar in formula and MOM. However, formula also contained Cyanobacteria, which was extremely rare in MOM. Despite being phylogenetically similar, MOM was largely dominated by *Staphylococcus*, while formula was largely dominated by *Lactococcus*, followed by *Streptococcus*, and *Pseudomonas*. This finding was not as surprising since formula is made from bovine milk, which is known to be abundant in *Lactococcus* and has also been reported to harbor *Streptococcus* and *Pseudomonas* (36). Additionally, an analysis of beta diversity showed a significant separation between formula and PDHM, revealing major differences in the principal constituents of their microbial communities likely due to inherent differences between bovine and human milk. *Lactococcus* is rarely reported as the causative agent of infections in humans and thus generally regarded as non-pathogenic. Nonetheless, there are a few dozen cases describing human infections caused by *Lactococcus,* and many of them involve patients who are immunocompromised (37). Since preterm infants have an immature immune system, they may be at an increased risk for infection associated with *Lactococcus* exposure.

It is important that we acknowledge one of the limitations of this work is the lack of qPCR results on the EN samples. While we did find notable differences in the microbial composition of EN samples based on relative abundance of the microbiome from 16S data, we recognize that quantifying the total bacterial load may highlight important differences in the absolute abundance of specific microbial taxa within these sample types. It is possible that differential taxa we have observed in different milk types using relative abundance may have similar absolute abundance, and taxa with similar relative abundance may have different absolute abundance. In future work, assessing the absolute quantities of microbes via qPCR in all EN samples would allow for a more comprehensive interpretation of the results.

In our longitudinal analysis of MOM during PPW 1–12, we observed that bacterial richness remained stable, while Shannon diversity tended to increase over time. Previous studies that examined longitudinal trends in alpha diversity of MOM reported mixed results, with three studies describing no significant changes and two studies describing a decrease in alpha diversity over time (29, 34, 38–40). At the phylum level, Proteobacteria tended to increase with time, whereas Firmicutes tended to decrease with time, which is consistent with the findings by Wan et al. (39). At the genus level, *Pseudomonas*, unclassified *Enterobacteria*, *Klebsiella*, *Lactococcus, Streptococcus*, *Acinetobacter*, and *Rothia* tended to increase with time, while *Staphylococcus*, *Finegoldia*, and *Peptoniphilus* tended to decrease with time. Asbury et al. reported similar trends in *Staphylococcus* and *Streptococcus* over time (34). In-line with our results, Gonzalez et al. reported that term MOM has a higher abundance of *Staphylococcus* in the early postpartum period and a higher abundance of *Pseudomonas* in the later postpartum period, but contrary to our results, they found a higher abundance of *Streptococcus* in the early postpartum period (40). Since *Streptococcus* and *Rothia* are common oral bacteria, it is possible that the increase in abundances that we observed over time reflects MOM collected following breastfeeding initiation (and thus, potential retrograde bacterial transfer), which tends to happen later in the postpartum period for preterm infants (35). Although little is known about what may be influencing temporal changes in other genera, our results suggest that such fluctuations do exist, which warrant further investigation. For instance, it is possible that changes in abundances of certain bacteria over time are not driven directly by maternal factors but instead occur in response to microbe-microbe interactions (e.g., *Staphylococcus* has been reported as inversely correlated with *Streptococcus* and *Lactobacillus*) (41). Future studies should incorporate a correlation network analysis to better understand bacterial interactions within human milk.

We identified several maternal factors that exhibited significant associations with the microbiota of MOM. *Streptococcus* was positively associated with birth GA, which supports the aforementioned theory that older preterm infants are more likely to breastfeed, and therefore, there is a higher probability of oro-mammary transfer of *Streptococcus* as gestational age increases (35). *Anaerococcus* was positively associated with maternal BMI at delivery. Since *Anaerococcus* has been shown to be enriched in the skin microbiota of overweight/obese individuals, it is possible that this bacterium would be present in higher abundances in the milk of women with a higher BMI (42). In MOM + NF samples, a higher abundance of *Lactococcus* was associated with a more alkaline milk pH. This was unexpected because *Lactococcus* is a lactic acid-producing bacterium and thus, we expected it to be associated with a more acidic milk pH. Additionally, it should be noted that *Lactococcus* does not normally colonize human tissues, leading us to hypothesize that it may have originated from NF, which likely contains *Lactococcus* since it is derived from bovine milk. The presence of *Lactococcus* in fortified milk may have similar implications for the health of preterm infants as mentioned above in the context of formula. More research is needed to assess the viability of specific bacteria and understand their role in overall milk biology.

Analogous to how gut microbiome has been described in terms of distinct “enterotypes,” several studies have attempted to identify major patterns in the milk microbial community (43). Based on our clustering analysis, MOM samples were separated into three distinct groups that we defined as putative “lactotypes.” Lactotype 1 was dominated by *Staphylococcus*, whereas lactotype 3 was dominated by *Pseudomonas*. Although it remains debated whether *Pseudomonas* is part of the core microbiota of human milk or represents a contaminant, it has been found in 50% of the studies included in a large systematic review (19). Lactotype 2 was characterized by a more mixed composition, with the four most abundant genera being *Staphylococcus*, *Enterococcus*, *Propionibacterium*, and *Streptococcus*. We found that lactotype 1 and lactotype 2 tended to appear in earlier PPW compared to lactotype 3, which tended to appear in later PPW. Biagi et al. reported three milk community types (MCTs), and similar to our lactotype 1, their MCT2 samples were characterized by the highest abundance of *Staphylococcus* and were collected earlier in lactation (35). Li et al. also reported three clusters, with Cluster 1 being driven by *Staphylococcaceae*, Cluster 2 by *Streptococcaceae*, and Cluster 3 by *Pseudomonadaceae,* which are similar to our lactotypes 1, 2, and 3, respectively (9). Although Moossavi et al. reported four clusters that did not fully align with our lactotypes, they did share a few dominant taxa including *Staphylococcaceae*, *Streptococcaceae*, and *Pseudomonadaceae*, suggesting that they could be among the major taxa shaping human milk microbiota (13).

Lactotypes 1 and 3 tended to have a longer latency period after rupture of membranes compared to lactotype 2. This is interesting in light of lactotypes 1 and 3 being dominated by a single genus (*Staphylococcus* and *Pseudomonas*, respectively). Since a prolonged latency period after ROM has been associated with a higher risk of chorioamnionitis, it may be possible that it also influences the bacterial community of the milk, although the exact mechanism behind selective proliferation of *Staphylococcus* and *Pseudomonas* is unclear (44). Antibiotic prophylaxis is used routinely during the intra-partum period, but the influence of antibiotics on the milk microbiota remains poorly understood. In our cohort, women with lactotype 3, which was dominated by *Pseudomonas,* were more likely to have received azithromycin and penicillin. These findings are similar to those of Asbury et al. who reported that prenatal penicillin exposure was associated with increased *Pseudomonas* (34). Interestingly, women with lactotype 2, which had a more mixed bacterial composition, were less likely to have received azithromycin and penicillin. In sum, our results suggest that the lack of exposure to these antibiotics is associated with a more diverse milk microbiota, which is supported by Asbury et al. who reported that prenatal macrolide exposure (of which azithromycin is a member) was associated with lower microbial diversity (34). Since antibiotics are known to enter milk in low concentrations, it is conceivable that they could directly alter its microbiota (45). Alternatively, the microbiota could be influenced indirectly by antibiotic-induced changes in the gut and skin microbiome (34).

In conclusion, our results suggest there are significant differences in the microbiota of EN. Additionally, our findings suggest the microbiota of MOM changes as a function of time and maternal factors. We also propose three lactotypes within MOM, which are associated with distinct microbial compositions and maternal factors. Taken together, our study sets the stage for future research aimed at advancing our understanding of the potential link between the microbiota of EN, gut colonization, and immune-mediated outcomes among preterm infants, which in turn, may facilitate the development of personalized nutritional interventions.

## MATERIALS AND METHODS

### Study population

We conducted a longitudinal retrospective observational study of preterm infants born between October 2018 and December 2019 (*n* = 72 infants and *n* = 65 mothers due to seven twin births). This study was approved by the Mass General Brigham Human Research Committee (protocol # 2016P001020) and performed at the NICU at Brigham and Women’s Hospital in Boston, MA. The study’s inclusion criteria were infants born at ≤ 34 weeks of gestation; exclusion criteria were infants who were expected to be transferred or who may not survive beyond 48 hours after birth. A total of 341 EN samples were used in our analyses: 83 MOM, 155 MOM + NF, 6 PDHM, 24 PDHM + NF, and 73 formula. The analyses that were limited to only MOM and MOM + NF samples were collected from 54 mothers during postpartum weeks 1–20 (238 samples) or postpartum weeks 1–12 (222 samples).

Maternal data were collected from electronic medical records. Dietary intake in the last month of pregnancy was assessed using the Dietary Screener Questionnaire developed by the National Cancer Institute. For women with twins, only twin “A” data were used in our analyses.

### Sample collection

Aliquots (5–10 mL) of EN samples were collected using a syringe prior to their administration to infants. MOM was expressed within 0–4 days of collection and stored at 4°C prior to collection. PDHM, which was obtained from HMBANA-certified Mothers’ Milk Bank Northeast (Newton Upper Falls, MA), was thawed within 0–2 days of collection and stored at 4°C prior to collection. MOM and PDHM that were fortified by nurses with nutritional fortifiers are referred to as MOM + NF and PDHM + NF, respectively. The fortification process followed standard clinical procedures, with NF including Similac Human Milk Fortifier Concentrated Liquid (Abbott, Abbott Park, IL), Liquid Protein Fortifier (Abbott, Abbott Park, IL), and MCT Oil (Nestle HealthScience, Bridgewater, NJ). Formula samples represent a range of ready-to-feed liquid formulas for preterm infants, such as Similac (Abbott, Abbott Park, IL) and Enfamil (Mead Johnson, Chicago, IL), that were stored at room temperature prior to collection. The nutritional composition of the formulas varied in response to infant’s nutritional needs by age and postpartum week but did not differ with respect to bioactive components (formulas fed to infants in this study did not contain any human milk oligosaccharides). For this reason, we reported the data generated from the formula samples as one group. All EN samples were frozen at −80°C within 4 hours of collection. A schematic of the experimental design and timeline is shown in Fig. S2.

### DNA isolation and 16S rRNA gene sequencing

We used standard procedures for bacterial DNA extraction and 16S rRNA gene sequencing. A brief summary is provided in Supplemental Materials and Methods.

### Decontamination

Using the R package “decontam” (version 1.18.0) with a threshold of 0.1, we identified and removed 22 potential contaminant operational taxonomic units (OTUs) that showed an increased prevalence in negative controls (19 extraction negative controls and 6 PCR negative controls) (46).

### Statistical analysis

Alpha diversity metrics were calculated with R package “vegan” (version 2.6–2) (47). Relative abundances were calculated by dividing the number of counts of each taxon by the total number of raw counts and then log2 transformed to improve normality. Dominant taxa were defined as those present in >20% of samples.

The Kruskal-Wallis rank sum test was used to examine differences in alpha diversity and abundances across EN samples. The Wilcoxon rank sum test was used to further examine the pairwise differences between EN types. For both tests, the *P* values were adjusted by false discovery rate (FDR) due to multiple comparisons, and adjusted *P* < 0.05 was considered statistically significant (48).

PCA was used to visualize dominant genera across EN types. PERMANOVA, a non-parametric permutation test based on Bray-Curtis distance matrices, was used to examine differences in microbial communities between EN types (49). The homogeneity of dispersion was assessed using the PERMDISP2 procedure (“betadisper*”* function in R package “vegan”), and only indices with homogeneous dispersion (*P* > 0.05) were tested (“adonis*”* function in R package “vegan”) (50).

A linear mixed model (LMM) was used to detect longitudinal trends in alpha diversity and bacterial abundance in MOM and MOM + NF groups. Initially, a full LMM was fitted where PPW (continuous), group, and their interaction term were treated as fixed effects and subject ID as a random effect. The interaction term was used to test whether the two groups had different slopes of PPW. If the interaction term was not significant (*P* > 0.05), it was removed, resulting in a reduced LMM.

To test the effect of group (MOM vs MOM + NF) on the bacterial abundance in each PPW, we used the same full and reduced LMM approach as above (except PPW was treated as categorical). The full LMM assumed the effect of group was different in each PPW, while the reduced LMM assumed it was the same. First, we tested the overall effect of group. Then, if the full model was selected, we further tested the differential abundance of the response variable between MOM and MOM + NF groups in each PPW. In Fig. 4D and 5D, only reduced models were chosen among the selected taxa with a significant group effect.

An LMM was used to analyze associations between maternal factors and bacterial abundances in MOM and MOM + NF groups where PPW (continuous), group, and maternal factors were treated as fixed effects and subject ID as a random effect. An interaction term between PPW and group was not used because it was insignificant (*P* > 0.05) in all models. For the correlation between *Lactococcus* and pH only, an interaction term between pH and group was added to fixed effects because we observed the group-wise slope difference when we reviewed results. The *F*-test on the effect of pH in the two groups was conducted separately for this correlation.

Dirichlet multinomial mixture clustering via R package “DirichletMultinomial” was used to identify lactotypes within MOM and MOM + NF samples at the genus level (51). This analysis included taxa present in >20% of samples and with a mean relative abundance >0.1%. Akaike information criterion was used to select optimal number of clusters. A principal coordinate analysis plot of the Jensen-Shannon divergence was used to visualize the distribution of bacterial genera (52). Lastly, a correlation analysis was conducted between lactotypes and maternal factors using the Kruskal-Wallis rank sum test if the outcome variable was continuous, or using the chi-squared test if it was categorical.

In each correlation analysis, samples that were missing maternal data were removed from the analysis. For all LMM models, *P* values were calculated from the *F*-test in R package “lmerTest,” which is based on Satterthwaite approximations to the degrees of freedom (53). The *P* values were adjusted by FDR with *P* < 0.1 (two-sided) considered as significant, unless otherwise specified.

## Data Availability

The data and source code that support this work are available upon request. The sequences have been uploaded and the accession number is PRJNA1035498.

## SUPPLEMENTAL MATERIAL

Supplemental material mbio.02106-23-s0001.docx Supplemental methods, Fig. S1 and S2, and Table S1.

ASM does not own the copyrights to Supplemental Material that may be linked to, or accessed through, an article. The authors have granted ASM a non-exclusive, world-wide license to publish the Supplemental Material files. Please contact the corresponding author directly for reuse.
