# Supplementary material for: The preterm human milk microbiota fluctuates by postpartum week and is characterized by gestational age and maternal BMI
Source: mBio. 2023 Nov 17;14(6):e02106-23. doi: 10.1128/mbio.02106-23 (PMC10746270; doi:10.1128/mbio.02106-23)
Supplement: Supplemental material — Supplemental methods, Fig. S1 and S2, and Table S1. [file mbio.02106-23-s0001.docx]

**Supplemental Materials and Methods**

***DNA Isolation***

A preliminary study was conducted to compare methodologies for isolating bacterial DNA from human milk based on three experimental variables: starting volume (2ml vs. 4ml), presence or absence of NF, and kit type (QiaAmp DNA Microbiome Kit, DNeasy PowerFood Microbial Kit, and DNeasy PowerSoil Pro Kit) (Qiagen, Hilden, Germany). These initial experiments were carried out in triplicates using aliquots from a single bottle of pooled preterm human milk and were subjected to the following analyses: sequencing read depth and species richness, alpha- and beta- diversity measures, and relative abundances (Figure S1). Since the DNeasy PowerSoil Pro Kit and the starting volume of 2ml produced the most consistent results while maintaining the greatest richness and diversity, this approach was used for this subsequent study as summarized below. Also, since the presence of NF did not make an appreciable change in the results, we included both unfortified and fortified samples in our study.

EN samples were randomized and DNA extraction was carried out in batches of 22. Briefly, EN aliquots (2ml) were thawed overnight at 4°C, then centrifuged at 15,000 x g at room temperature to separate fat and cells from the aqueous portion. A sterile, cotton-tipped swab was used to remove the top fat layer and the “skimmed” supernatant was transferred into another tube for later pH measurement, leaving a bacterial pellet at the bottom of the original tube. Bacterial DNA was isolated from the pellets using the DNeasy PowerSoil Pro Kit (Qiagen, Hilden, Germany) according to the manufacturer’s protocol without modifications. Two samples containing 2ml of sterile HyClone water (Cytiva, Marlborough, MA) were used as negative extraction controls for each batch.

***pH Measurement***

The tubes containing EN supernatant (described above) were submerged in a water bath at 37°C to simulate a routine NICU nursing practice of warming feedings prior to administration. The pH was measured using a digital pH-meter (HI5221, Hanna Instruments, Smithfield, RI) equipped with an extended-length glass electrode with a micro bulb (HI1093B, Hanna Instruments, Smithfield, RI) and an automatic temperature compensation. The pH meter was calibrated daily with pH 4.0 and 7.0 buffers following the manufacturer's instructions.

***16S rRNA Gene Sequencing and Data Processing***

Polymerase Chain Reaction (PCR) was performed on the V1-3 region of the 16S rRNA gene using the primer of 27F (5’- AATGATACGGCGACCACCGAGATCTACACNNNNNNNNACACTCTTTCCCTACACGA-3’) and 534R (5’-CAAGCAGAAGACGGCATACGAGATNNNNNNNNGTGACTGGAGTTCAGACGTGTGCTCTTCCGATCTATTACCGCGGCTGCTGG-3’), where N’s contain a unique 8bp index for each sample. Amplicon pools were sequenced on the Illumina MiSeq sequencing platform to generate 300bp, paired-end reads. Microbial Community Standards were included as positive sequencing controls and purified water as sequencing negative controls. Sequencing data were processed using the UPARSE pipeline (usearch v8.0.1517) clustered into operational taxonomic units (OTUs). Taxonomy was assigned using the Ribosomal Database Project (RDP) classifier v2.2 database. Sequencing reads from all EN sample were rarified to the lowest read counts (4231).

**Figure S1.** Results from a preliminary study conducted to identify an optimal method for isolating bacterial DNA from human milk samples. **A)** Sequencing read depth and species richness stratified by kit type, **B)** alpha diversity measures (Chao1 and Shannon) stratified by kit type, **C)** NMDS of beta diversity stratified by fortification status and kit type, and **D)** mean relative abundance of dominant genera stratified by kit type, fortification status, and starting volume.

**
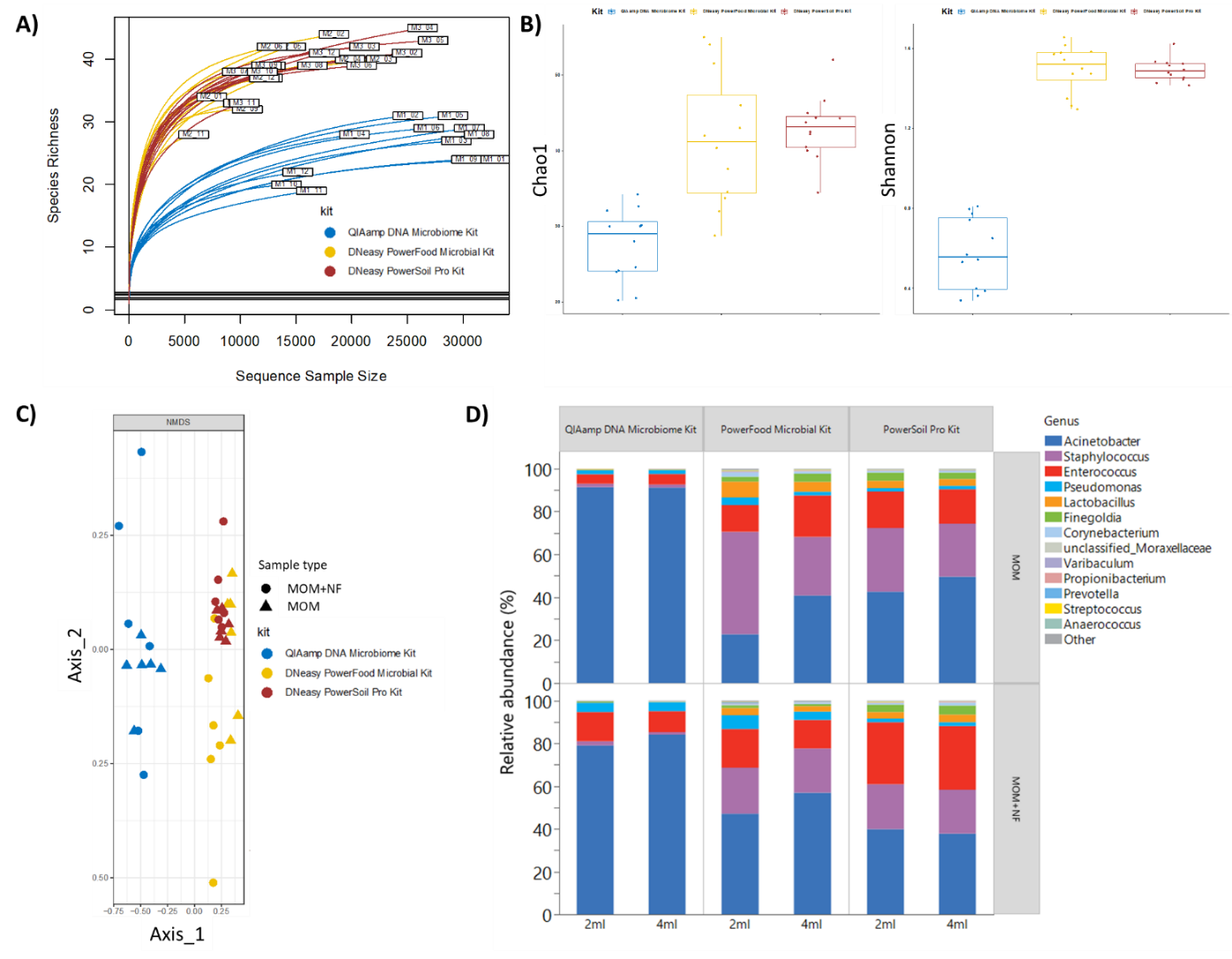
**

**Abbreviations:** MOM – mother’s own milk, NF – nutritional fortification.

**Figure S2.** Experimental design and study timeline.


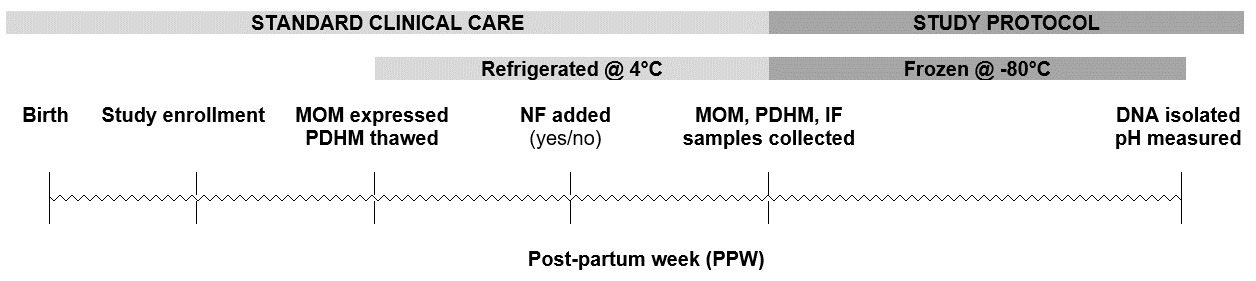


**Abbreviations:** MOM – mother’s own milk, PDHM – pasteurized donor human milk, NF – nutritional fortification, IF – infant formula.

**Table S1.** Sample mean relative abundance of taxa from the three lactotypes (%).

| **Genus** | **Lactotype 1** | **Lactotype 2** | **Lactotype 3** |
| --- | --- | --- | --- |
| *Staphylococcus* | 93.20 | 46.51 | 5.81 |
| *Pseudomonas* | 0.49 | 3.06 | 87.31 |
| *Propionibacterium* | 1.61 | 7.71 | 0.07 |
| *Streptococcus* | 0.56 | 7.71 | 1.55 |
| *Enterococcus* | 0.59 | 8.08 | 0.15 |
| *Corynebacterium* | 1.79 | 6.54 | 0.37 |
| *Acinetobacter* | 0.07 | 5.81 | 0.43 |
| *Prevotella* | 0.12 | 2.54 | 0.00 |
| *Finegoldia* | 0.40 | 1.81 | 0.01 |
| *unclassified_Enterobacteriaceae* | 0.02 | 0.78 | 0.11 |
| *Klebsiella* | 0.06 | 0.52 | 0.34 |
| *Anaerococcus* | 0.02 | 0.53 | 0.00 |
| *Actinomyces* | 0.07 | 0.50 | 0.00 |
| *Lactococcus* | 0.18 | 0.35 | 0.01 |
| *Peptoniphilus* | 0.03 | 0.46 | 0.00 |
| *Veillonella* | 0.14 | 0.15 | 0.01 |
| *Rothia* | 0.01 | 0.19 | 0.02 |
| Others | 0.64 | 6.75 | 3.82 |
